# 58 Positioning Pediatric Acute Palmar Burn Patient: Experience with Soft Cast and Thermoplastic Splint

**DOI:** 10.1093/jbcr/iraf019.058

**Published:** 2025-04-01

**Authors:** Hilary Smith-Chong, Jessica Willoughby, Lori Connolly

**Affiliations:** Shriners Children’s – Boston; Shriners Children’s – Boston; Shriners Children’s – Boston

## Abstract

**Introduction:**

Hand burns are a leading cause of functional impairment after burn injury. Palmar burn injuries crossing joints or creases are high risk for contracture. Research supports early rehabilitation, stretching and orthotic management. Standard practice at our facility is every other day dressing change and positioning in thermoplastic splint (TS), though trials exist maintaining palmar extension. While evidence supports TS, mounting research backs use of soft casting (SC) and dressing change every 3-7 days. Currently no consensus exists on ideal dressing type, cost factors, or timing of dressing change, though both TS and SC are confirmed safe and effective. We aim to compare TS to SC and develop guidance on ideal positioning based on individual patient/family characteristics and needs.

**Methods:**

A prospective observational cohort study using a convenience sample from a single pediatric burn center. Eligibility included patients aged 6 months to 18 years with palmar burn or friction injury crossing joints or palmar creases of hand. Therapist provided education on two different positioning devices and parent/guardian chose preferred intervention. Data collected at each dressing change, wound closure, and 1-month follow-up visit included need to replace positioning device, wound assessment, signs of infection, ROM assessment, presence of auto-release and photographs. Hand span/hand length measurements obtained at wound closure and 1-month follow-up. Parent satisfaction survey was also conducted at 1-month follow-up.

**Results:**

Goal recruitment is 50 subjects, with 35 subjects enrolled and 22 have completed study protocol, allowing general descriptive statistics to be performed. Of the subjects, 20 had SC and 16 had TS for positioning. Average day to wound closure for TS was 18.75 vs SC at 14.54 days. Both groups had equal incidence of auto-release. No infections reported after initial visit in either group. To date, 22 parent satisfaction surveys are complete. Responses show 53% of SC families reported missed work days vs 88% of TS families, with TS families reporting more total days missed. All respondents strongly agreed to receiving appropriate education on positioning options. Majority of SC families responded strongly agree with comfort managing cast at home, with 7% responding somewhat agree. TS group response regarding comfort level varied (66% strongly agree, 22% somewhat agree, 11% somewhat disagree). Satisfaction level with treatment of child’s hand burn was equal with majority stating extremely satisfied and 1 from each group reporting somewhat satisfied.

**Conclusions:**

Preliminary data reinforces that both positioning interventions are safe and effective options in practice. Satisfaction survey results to date demonstrate trends to support informed decision-making when choosing a positioning intervention.

**Applicability of Research to Practice:**

Research supports development of therapeutic guidelines in treatment of pediatric palmar burns

**Funding for the Study:**

N/A

